# Evaluation of sexual function in patients submitted to ureteroscopic procedures

**DOI:** 10.1590/S1677-5538.IBJU.2014.0353

**Published:** 2015

**Authors:** Bilal Eryildirim, Murat Tuncer, Cahit Sahin, Ugur Yucetas, Kemal Sarica

**Affiliations:** 1 Dr. Lütfi Kirdar Training and Research Hospital Urology Clinic, Istanbul, Turkey; 2 Istanbul Training and Research Hospital Urology Clinic, Istanbul, Turkey

**Keywords:** Ureteroscopy, Questionnaires, Urinary Tract Infections

## Abstract

**Objective:**

We aimed to evaluate the possible effects of ureteroscopic procedures on the sexual function of both genders.

**Materials and Methods:**

A total of 102 sexually active cases (60 male, 42 female) undergoing ureteroscopic procedures were included in this study. Sexual function has been evaluated in detail by using International Index of Erectile Function (IIEF) in male and Female Sexual Function Index (FSFI) forms in female cases both before and 1-month after the procedures. Pre-and postoperative data were evaluated in a comparative manner.

**Results:**

The pre-and postoperative mean IIEF scores were 57.86±2.26 and 54.57±2.48 (p=0.19) in males and the mean FSFI scores were 13.58±1.46 and 14.46±1.52 (p=0.41), respectively in females. Evaluation of these values showed that regarding the effects of this procedure on male cases although the total scores for sexual function were not influenced it was observed a significant reduction in the intercourse satisfaction sub-domain (IIEF-IS) in males (p<0.05). In female cases however, unlike the male cases no statistically significant alterations with respect to these scores were noted (p=0.418).

**Conclusion:**

Ureteroscopic interventions could have some adverse effects on the sexual function particularly in male cases. However, it is clear that further prospective studies in both genders with large population of cases are certainly needed in order to outline this unresolved but important subject.

## INTRODUCTION

Ureterosocopic procedures are commonly applied both in the diagnosis as well as in the management of ureteral pathologies among which the removal of stones is the commonest. Although the procedure is accepted as minimally invasive some certain complications and related complaints can be noted in a certain percent of the cases. However, as a result of the technologic improvements, ureterorenoscopes became thinner and with the advanced visualization, the success rates have increased and the complication rates decreased. However, despite a successful procedure, lower urinary tract symptoms can be observed due to edema, coagulum and stone fragments which may cause a temporary urinary obstruction. Additionally, published limited data showed that these bothersome lower urinary tract symptoms may further affect the sexual function of the treated patients.

In this study, we aimed to evaluate the possible effects of ureterorenoscopic procedures on the sexual functions of both genders.

## MATERIALS AND METHODS

One hundred and two sexually active patients (60 male, 42 female) undergoing diagnostic and/or therapeutic ureterorenoscopy for ureteral stone(s) were included in this study program. Local ethic approval was obtained from our Hospital Ethics Committee and all cases were well informed about the procedure in detail with and informed consent obtained prior to the intervention.

Cases with acute urinary infection, uro-genital system tumor, previous urogenital system surgery, neurogenic bladder, bladder stone, overactive bladder, chronic prostatitis, benign prostatic hyperplasia and previous pelvic injuries were excluded from the study. Additionally, patients with ureteral stricture diagnosed during the procedure, and patients in whom a mucosal trauma was induced and/or a double j stent was placed (due to residual fragments, stone migration and edema formation) were excluded from the study.

In addition to a detailed anamnesis, a careful physical examination was done and biochemical exams included renal functional tests, urine analysis and urine culture-sensitivity tests. During radiological evaluation of the urinary system although a plain abdominal film (KUB) and ultrasonography of the urinary system were performed in all cases, non-contrast CT evaluation was performed when needed. To evaluate the sexual function in both genders, “International Index of Erectile Function” (IIEF) questionnaire form has been used in men and a “Female Sexual Function Index” (FSFI) questionnaire form was utilized in women. All cases responded the questionnaries in clinical conditions and they have filled the relevant forms in the department (1, 2). This procedure was repeated after 4-weeks following the ureteroscopic procedures.

All ureteroscopy procedures were performed under general anesthesia by using a semirigid 8 Fr ureteroscope (Karl Storz, Tuttlingen, Germany). All cases received 1gram of Cephazolin injection for prophylaxis prior to the procedure. Stone fragmentation was accomplished by using holmium-YAG laser (with 550 micron fiber).

Statistical analysis of the obtained scores was made by using Graphpad prism 5.0 program. In addition to the descriptive statistical methods (mean, Standard deviation), paired t test was used for the comparison of the quantitative data during this analysis. The results were assessed at 95% confidence interval and a p value of<0.05 was accepted as significant.

## RESULTS

### Evaluation of sexual function in males

**Figure 1:**
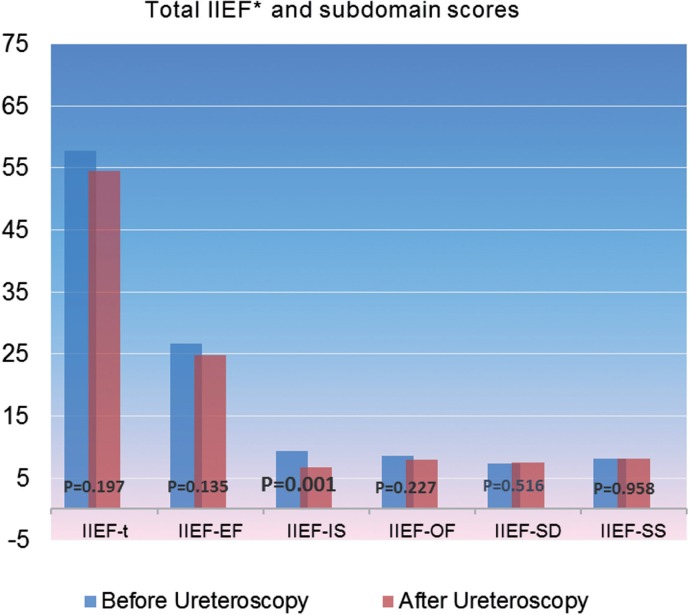
Total IIEF and subdomain scores before and after ureteroscopy.

Mean age of the males evaluated was 42.07±1.83 year (27-67 year). Evaluation of the total IIEF (IIEF-t) scores in these cases before and after the procedure a mean score of 57.86±2.26 prior to the intervention and a mean score value of 54.57±2.48 after 4-weeks respectively (p=0.19) did not reveal statistically significant change in IIEF-t scores. However, further evaluation of the IIEF-t scores in a subdomain based manner sub-domains including erectile function (IIEF-EF), intercourse satisfaction (IIEF-IS), orgasmic function (IIEF-OF), sexual desire (IIEF-SD) and overall satisfaction (IIEF-SS)), it was observed a significant reduction in the intercourse satisfaction subdomain (IIEF-IS) in male cases. This finding showed us that although the total scores for sexual function may not be influenced after this procedure, male patients can report a reduction in intercourse satisfaction, a subdomain, that should be accepted as important for this gender. Mean scores for IIEF–t and subdomains score values of the males noted before and after the procedure are presented in Table-1 and Figure-1.

**Table 1:** Total IIEF and subdomains scores before and after ureteroscopy in men.

			p
	Before URS	After 4-weeks	
IIEF-t	57.86±2.26	54.57±2.48	0.197
IIEF-EF	26.61±1.15	24.77±1.29	0.135
IIEF-IS	9.32±0.46	6.66±051	0.001
IIEF-OF	8.54±0.37	7.89±0.44	0.227
IIEF-SD	7.34±0.27	7.48±0.26	0.516
IIEF-SS	8.04±0.32	8.05±0.29	0.958

### Evaluation of sexual function in females

Mean age value of the females evaluated was 43.67±2.14 year (26-65 year). Evaluation of the total FSFI-t scores in these cases before and after the procedure [(a mean score of 13.58±1.46 prior to the intervention and a mean score value of 14.46±1.53 after 4-weeks respectively (p=0.418)] did not reveal any statistically significant change in FSFI-t scores.

**Figure 2:**
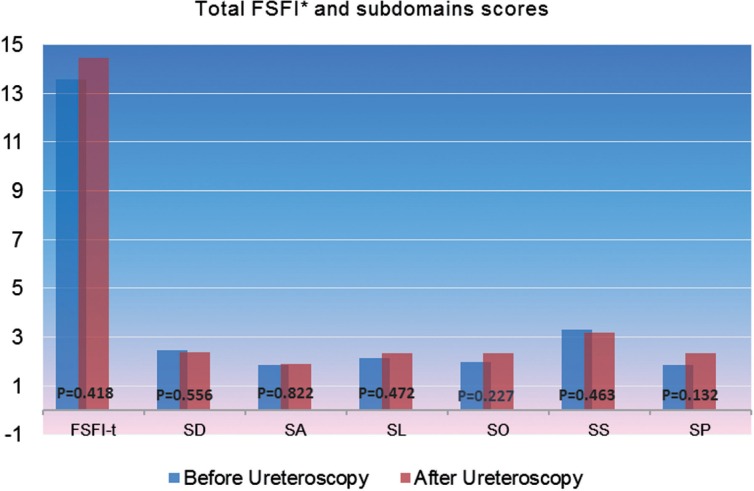
Total FSFI and subdomain scores before and after ureteroscopy.

Additionally unlike the male cases further evaluation of the FSFI-t scores, in a subdomain based manner (subdomains including sexual desire (FSFI-SD), sexual arousal (FSFI-SA), sexual lubrication (FSFI-SL), orgasm (FSFI-SO), general satisfaction (FSFI-SS) and pain (FSFI-SP) did not again demonstrate any statistically significant alteration with respect to these subdomain scores. FSFI-t and subdomains values of the females obtained before and after the procedure are presented in Table-2 and Figure-2.

**Table 2:** Total FSFI and subdomains scores before and after ureteroscopy in women.

			p
	Before URS	After 4-weeks	
FSFI-t	13.58±1.46	14.46±1.53	0.418
FSFI-SD	2.45±0.18	2.36±0.16	0.556
FSFI-SA	1.85±0.26	1.90±0.25	0.822
FSFI-SL	2.12±0.31	2.34±0.33	0.472
FSFI-SO	1.97±0.30	2.35±0.34	0.227
FSFI-SS	3.31±0.31	3.20±0.31	0.463
FSFI-SP	1.87±0.30	2.35±0.34	0.132

## DISCUSSION

Endourological procedures were introduced in the clinical practice in the 1980s and since then they have become widely accepted as reliable methods for ureteral stone treatment with few complications (3, 4). Ureterosocopic procedures are commonly applied both in the diagnosis as well as the management of ureteral pathologies among which the removal of stones is the commonest. Compared with shockwave lithotripsy (SWL), as a minimal invasive alternative, currently with the clinical introduction and use of thinner and fine instruments it is possible a detailed exploration and appropriate management of all stones located in different parts of the ureter with higher success rates and minimal trauma (5–8). Despite their minimally invasive nature and high success rates obtained, possible adverse effects of these procedures on the quality of life as well as on the sexual functions of the cases treated have not been subjected to the studies in detail so far.

Sexual dysfunction is a condition that can be frequently seen after some certain urologic operations among which radical retropubic prostatectomy and radical cystoprostatectomy are the more usual procedures that cause this problem in a considerable percentage of patients (9). However, published data did also show that this problem may even be encountered after some relatively minor interventions. For example Akbal et al. reported that temporary ED at a rate of 11.6% could be noted in patients with normal erectile function undergoing saturation biopsies of the prostate (10). To support this observation several studies showed that sexual functions may be negatively affected in both genders after placement of ureteral catheter for certain pathologies (11, 12). In a recent study focusing on the possible relationship between stent placement and sexual dysfunction Sighinolfi et al. used both the IIEF and FSFI questionnaires in a series of 50 patients and they were able to show that a stent-related impairment in sexual lives of the treated cases could be anticipated after this procedure (13). In our recently published original study we were able to show the negative effects of ureteral stent placement on the sexual life of cases that could be explained by the presence of lower urinary tract symptoms, flank pain, anxiety and sleep disturbance after the procedure (14).

Apart from the procedures mentioned above and well evaluated on this aspect the possible effects of commonly performed endourologic procedures have not been determined (15). There is only one study published so far in the literature evaluating the possible adverse effects of endourologic procedures on sexual functions and in this original study Sofer et al. aimed to evaluate the effects of endourologic procedures on sexual function only in male cases and they found that these procedures may cause a temporary post-operative sexual dysfunction, which recovers within 3 months (16). However, in that study a DJ stent has been inserted in 66% of patients which may also be another possible factor responsible for this temporary deterioration.

According to our study, male patients can experience reduction in intercourse satisfaction, a subdomain, that should be accepted as important for this gender. However, in female cases, further evaluation of the FSFI-t scores in a subdomain based manner did not demonstrate any statistically significant alteration with respect to these scores. In the light of the findings we have obtained, we may comment that the limited but important changes in male cases could be originated from lower urinary system symptoms resulting from neuronally-rich trigon mucosa irritation, anxiety, insomnia and depression leading to sexual dysfunction.

Our study has certain limitations. First of all the number of the cases included into the study program is limited. Also, long-term follow-up data is lacking in both groups. Although we noted no significant change with respect to the total scores in males after 1-month evaluation we believe that alterations observed in intercourse satisfaction during this period should also be evaluated and followed in a long-term follow-up. However, taking the highly limited data published in the literature so far into account we believe that our preliminary data will be valuable and contributive on this aspect.

## CONCLUSION

Our results and very limited data published in the literature show that a commonly performed procedure (ureteroscopic intervention) could have some adverse effects on the sexual function particularly in male cases. However, it is clear that further prospective studies in both genders with large population of cases are certainly needed in order to outline this unresolved but important subject.
